# Overexpressed FATP1, ACSVL4/FATP4 and ACSL1 Increase the Cellular Fatty Acid Uptake of 3T3-L1 Adipocytes but Are Localized on Intracellular Membranes

**DOI:** 10.1371/journal.pone.0045087

**Published:** 2012-09-14

**Authors:** Tianzuo Zhan, Margarete Poppelreuther, Robert Ehehalt, Joachim Füllekrug

**Affiliations:** Molecular Cell Biology Laboratory, Internal Medicine IV, University of Heidelberg, Heidelberg, Germany; Université de Genève, Switzerland

## Abstract

Long chain acyl-CoA synthetases are essential enzymes of lipid metabolism, and have also been implicated in the cellular uptake of fatty acids. It is controversial if some or all of these enzymes have an additional function as fatty acid transporters at the plasma membrane. The most abundant acyl-CoA synthetases in adipocytes are FATP1, ACSVL4/FATP4 and ACSL1. Previous studies have suggested that they increase fatty acid uptake by direct transport across the plasma membrane. Here, we used a gain-of-function approach and established FATP1, ACSVL4/FATP4 and ACSL1 stably expressing 3T3-L1 adipocytes by retroviral transduction. All overexpressing cell lines showed increased acyl-CoA synthetase activity and fatty acid uptake. FATP1 and ACSVL4/FATP4 localized to the endoplasmic reticulum by confocal microscopy and subcellular fractionation whereas ACSL1 was found on mitochondria. Insulin increased fatty acid uptake but without changing the localization of FATP1 or ACSVL4/FATP4. We conclude that overexpressed acyl-CoA synthetases are able to facilitate fatty acid uptake in 3T3-L1 adipocytes. The intracellular localization of FATP1, ACSVL4/FATP4 and ACSL1 indicates that this is an indirect effect. We suggest that metabolic trapping is the mechanism behind the influence of acyl-CoA synthetases on cellular fatty acid uptake.

## Introduction

The lipid metabolism of adipose tissue plays an important role in health and is involved in the pathogenesis of several diseases [1], [2]. The cellular uptake of free fatty acids is a central step within lipid metabolism as it enables the synthesis of neutral lipids, and also provides building blocks for lipid membranes and substrates for beta-oxidation. A number of proteins have been identified that are involved in the uptake process [3], but the mechanism of their contribution is controversially discussed [4], [5], [6], [7].

One family of proteins involved in fatty acid uptake is the acyl-CoA synthetase family. They are highly conserved enzymes which catalyze the ATP-dependent esterification of long chain fatty acids (LCFAs) with coenzyme A, transforming them into activated intermediates for either beta-oxidation or the biosynthesis of lipids [8]. Fatty acid transport protein 1 (FATP1), very long chain acyl-CoA synthetase 4 (ACSVL4/FATP4) and long chain acyl-CoA synthetase 1 (ACSL1) are the predominant acyl-CoA synthetases in adipocytes [9], [10], [11]. It is debated whether the FATP family proteins directly mediate fatty acid uptake by transport [4], [12] or by vectorial acylation/intracellular metabolism. A key observation was that ACSVL4/FATP4 increased fatty acid uptake even when it was localized to the endoplasmic reticulum (ER) of epithelial and muscle cells, suggesting it does not transport fatty acids across the plasma membrane [13], [14]. Metabolic trapping/vectorial acylation of fatty acids as acyl-CoA derivatives has been proposed to be the mechanism behind this enzyme-driven fatty acid uptake [7], [13], [15].

Insulin increases the fatty acid uptake of adipocytes, and FATP1 was proposed to mediate this effect [11]. This observation was supported by subsequent experiments with primary adipocytes from FATP1 knockout mice which showed no increase of fluorescent fatty acid uptake upon insulin stimulation [16]. Originally, it was assumed that FATP1 is translocated from intracellular compartments to the plasma membrane upon insulin treatment, analogous to GLUT4 [11], [16]. However, this has been questioned by recent publications which either did not observe a translocation [17] or only for a minor fraction of total FATP1 [18]. Also, the localization of FATP1 is still unresolved, since it was also reported to be on mitochondria [19], [20] and the Golgi apparatus [21].

ACSVL4/FATP4 was initially reported to be the major intestinal fatty acid transporter [22]. Knockdown of ACSVL4/FATP4 in 3T3-L1 adipocytes resulted in increased basal lipolysis and reduced cellular triglyceride content [18]. The intracellular localization of ACSVL4/FATP4 has been demonstrated for a number of different model systems [13], [14], [18], [23]. However, other studies proposed a localization of ACSVL4/FATP4 on the plasma membrane [11], [22], [24]. While no change in fatty acid uptake rate after insulin stimulation was found for ACSVL4/FATP4 knockdown adipocytes [18], an increase of uptake was observed in several ACSVL4/FATP4 overexpressing cell lines [13], [14], [22], [23].

ACSL1 is quantitatively the most abundant ASCL in adipocytes and its expression is highly increased during differentiation [9], [25], [26]. Knockdown of ACSL1 in 3T3-L1 adipocytes did not effect fatty acid uptake rate, but increased basal lipolysis [27], while adipocyte-specific knock-out of ACSL1 reduced beta-oxidation rates [28]. ACSL1 was found on different cellular compartments, including plasma membrane [29] and lipid droplets [29], [30] in adipocytes, ER and mitochondria-associated membranes in hepatocytes [31], and on mitochondria [13], [32].

In this study, we investigated the subcellular localization of FATP1, ACSVL4/FATP4 and ACSL1 in 3T3-L1 adipocytes by stable overexpression. We found that FATP1 and ACSVL4/FATP4 share a distinct intracellular localization which corresponds to the ER, while ACSL1 was localized primarily on mitochondria. The intracellular localization of all three proteins was sufficient to enhance fatty acid uptake. Insulin increased the uptake of fluorescent fatty acids in FATP1 and ACSVL4/FATP4 overexpressing adipocytes without changing the intracellular localization of both proteins. Thus, we could demonstrate that acyl-CoA-synthetases located intracellularly are sufficient to drive basal and insulin-stimulated fatty acid in 3T3-L1 adipocytes.

## Methods

### Antibodies

Antibodies used were obtained from the following sources: rabbit anti-FATP4 was generated as described earlier [13], rabbit anti-FATP1 was kindly provided by David Bernlohr (University of Minnesota, MN, USA), mouse monoclonal anti-GLUT4 was from Santa Cruz Biotechnologies (sc-53566, Heidelberg, Germany), mouse monoclonal anti-CD36 from Abcam (ab23680, Cambridge, UK), mouse anti-FLAG M2 from Sigma (St. Louis, MO), mouse anti-Na+/K+-ATPase from ABR Affinity BioReagents (A3-928, Golden, CO), rabbit anti-calnexin from Assaydesigns (SPA-865, Ann Harbor, MI), rabbit anti-VDAC1 from Abcam (ab15895, Cambridge, UK) and donkey anti rabbit/mouse coupled to Cy3, Cy2 or HRP from Jackson ImmunoResearch (West Grove, PA). Rabbit anti AMPKα (D63G4) and rabbit anti phospho-AMPKα (T172; 40H9) were from Cell Signaling (Danvers, MA).

### Cell Culture

3T3-L1 fibroblasts from ATCC (CL-173) were kindly provided by Susanne Mandrup (University of Southern Denmark, Denmark) and Christoph Thiele (University of Bonn, Germany) and cultured in Dulbecco’s modified Eagle’s medium with 4.5 g/L glucose (DMEM; Invitrogen, Karlsruhe, Germany), 10% fetal calf serum (Biochrom, Germany), 8 mg/L pantothenic acid, 8 mg/L D-biotin, 100 U/ml penicillin/streptomycin and 1% GlutaMax (Invitrogen, Karlsruhe, Germany). Phoenix-gp cells were grown in Dulbecco’s modified Eagle’s medium containing 4.5 g/l glucose, 10% fetal calf serum, 1% GlutaMax and 100 U/ml penicillin/streptomycin under standard tissue culture conditions.

### Adipocyte Differentiation

3T3-L1 fibroblasts were differentiated as described previously [33]. In brief, differentiation was induced in post-confluent 3T3-L1 fibroblasts with medium containing 500 mM 3-isobutyl-1-methylxanthine, 5 mM dexamethasone and 5 mg/ml insulin (Invitrogen, Karlsruhe, Germany) (day 0). On days 2–3, the medium was replaced by fresh medium containing 5 mg/ml insulin. After days 5–6, the medium was replaced every 2 days with fresh standard medium until maturation. Adipocytes were used 8–12 days post-induction for experiments.

### Plasmids for Stable Expression

The retroviral vector pRJ is derived from the moloney murine leukemia retrovirus based plasmid pQCXIP (BD Biosciences, Heidelberg, Germany) and contains a modified multiple cloning site [14]. FATP4.pRJ has been described previously [14]. FATP1.pRJ was derived by multiple subcloning steps from an EGFP-C1 plasmid (Clontech, Mountain View, CA, USA) containing mouse FATP1 (kindly provided by T. Hermann, University of Heidelberg, Germany). Rat ACSL1-FLAG.pRJ was generated by digestion of C-terminal FLAG-tagged ACSL1.pcDNA3 [34], kindly provided by R. Coleman (University of North Carolina, NC, USA), with BamHI, NotI and ligation into pRJ.

### Generation of Stably Expressing 3T3-L1 Cell Lines

The generation of infectious pseudotyped retroviral particles was essentially done as described [35]. In brief, Phoenix-gp packaging cells were cotransfected with VSV-G and one of the following plasmids FATP1.pRJ, FATP4.pRJ, ACSL1-FLAG.pRJ or the control vector pRJ. The phoenix supernatant, containing the replication incompetent retroviral particles, was harvested every 24 h, up to 6 days after transfection, and used to infect dividing 3T3-L1 fibroblasts. Two days after infection, selection was initiated by incubating cells with 8 µg/ml puromycin for 24 h. The cells were then allowed to recover for 24 h in fresh medium without antibiotics, followed by 24 h incubation with 8 µg/ml puromycin. Stably expressing cells were used only up to five passages after retroviral transduction.

### Expression Analysis by Quantitative Real-time PCR

Total RNA was extracted with High Pure RNA Isolation Kit (Roche, Mannheim, Germany) and reverse transcription was performed with Transcriptor First Strand cDNA Synthesis Kit (Roche, Mannheim, Germany) using hexanucleotides for random priming.

The mRNA levels of FATP1 (NM_011989.4) and ACSVL4/FATP4 (NM_011977.2) were determined by efficiency corrected relative quantification on an Applied Biosystems 7500 Fast Real-Time PCR System (Foster City, CA), using SYBR Green (Power SYBR Green Master Mix; Roche, Mannheim, Germany) for detection. The quantity of each mRNA was obtained by using a calibration curve derived from five subsequent dilutions of the corresponding plasmids. The quantities were normalized to the quantities of general transcription factor 2b (Gtf2b) (NM_145546.1), a housekeeping gene in adipocytes [36]. Real-time PCR primers used were verified to give a single product by melting curve analysis.

Primers (5′ to 3′):

ACSVL4/FATP4 GTGAGATGGCCTCAGCTATC, GAAGAGGGTCCAGATGCTCT.

FATP1 TCACTGGCGCTGCTTTGGTT, TAGCCGAACACGAATCAGAA.

Gtf2b GTTCTGCTCCAACCTTTGCCT, TGTGTAGCTGCCATCTGCACTT.

### Oleate Uptake

3T3-L1 adipocytes were starved in serum-free medium containing 1% bovine serum albumin (BSA). After 3 h, the medium was removed and the cells were washed with Krebs Ringer HEPES (KRH) buffer (120 mM NaCl, 4.7 mM KCl, 2.2 mM CaCl_2_, 10 mM HEPES, 1.2 mM KH_2_PO_4_, 1.2 mM MgSO_4_, pH 7.4). Then, serum-free medium containing defined concentrations of [^3^H]-oleic acid (ART-198; Biotrend Chemikalien GmbH, Cologne, Germany) bound to fatty acid free BSA in a specific ratio was added (specific activity 0.5–1 Ci/mol). After 5 min, 60 min or 3 h, the labeling mix was removed and the uptake was stopped by washing the cells 2x with ice-cold PBS containing 0.5% BSA and then 2x with ice-cold PBS. The cells were then lysed with 1M NaOH and aliquots of each lysate were used for scintillation counting in a Beta-Counter LS 6500 (Beckman-Coulter, CA). Protein concentration was measured by Bradford assay.

### Fluorescent Fatty Acid Uptake and Flow Cytometric Analysis

Fluorescent Bodipy fatty acids are very long chain fatty acid analogues and have been frequently used to measure cellular fatty acid uptake [11], [13]. We used two fatty acid species with different carbon chain length, Bodipy 500/510 (FA_By12_; 4,4-difluoro-5-methyl-4-bora-3a,4a-diaza-*s*-indacene-3-dodecanoic acid) and Bodipy FL_C16_ (FA_By16_; 4,4-difluoro-5-methyl-4-bora-3a,4a-diaza-*s*-indacene-3-hexadecanoic acid; both from Invitrogen, Carlsbad, CA), to quantify uptake via flow cytometric analysis (FACS). Adipocytes were serum-starved for 3 h and incubated in serum-free medium with or without 1 µg/ml insulin for 18 min. Then Bodipy fatty acids bound to BSA (final concentration: 2 µM FA_By12_/FA_By16_ and 2 µM BSA) were added. After 2 min, the labeling mix was removed; the cells were washed with ice-cold PBS and detached using trypsin/EDTA. After two cycles of washing with PBS and pelleting (5 min at 4°C and 1000×g), the cells were fixed with 4% paraformaldehyde (PFA) for 15 min at room temperature and then washed 2x with PBS. Fluorescence was measured in a Becton Dickinson FACSCalibur and analyzed with CellQuest Pro software (BD Biosciences, Germany). Values correspond to the geometric mean of all gated cells.

### Deoxyglucose Uptake

3T3-L1 adipocytes were starved for 3 h in serum-free medium containing 1% bovine serum albumin (BSA). The cells were then washed and, if indicated, stimulated with 1 µg/ml insulin in KRH buffer for 20 min at 37°C. Glucose transport was measured by adding [3H]-2-deoxy-D-glucose (NET-549, PerkinElmer, Waltham, MA) (final concentration: 0.1 mM 2-deoxy-D-glucose [DOG], 1 µCi/ml) for 10 min. Uptake was stopped by immediately removing the labeling mix and washing 4x with ice-cold PBS. Radioactivity and protein measurement were done as described for oleate uptake.

### Transfection with Nucleofector™

3T3-L1 adipocytes were transiently transfected with the following plasmids: OCT-GFP.pcDNA3 (N-terminus of ornithine carbamyl transferase [37]) kindly provided by Heidi McBride, University of Ottawa, Canada), ER-RFP (full-length human Sec61-β followed by mRFP [33] kindly provided by Christoph Thiele, University of Bonn, Germany). Tom20-GFP contains amino acids 1–30 of the human outer mitochondrial membrane receptor Tom20 followed by EGFP (Clontech), and was provided by Daniela Lehnen, University of Heidelberg, Germany.

In brief, adipocytes on day 6–8 of differentiation were detached with trypsin and suspended in standard medium containing 4% glycerol. The cells were centrifuged for 8 min at 8,000 g and room temperature, and then resuspended in medium containing 4% glycerol. An aliquot of this cell suspension containing 2×10^6^ cells was taken, centrifuged and the remaining medium was completely removed. This pellet was resuspended in 100 µl Nucleofector solution L (Amaxa, Cologne, Germany) containing 2–3 µg DNA. Transfection was performed in a Nucleofector (Amaxa, Cologne, Germany) with the default program A-033. After transfection, 500 µl of fresh medium was immediately added. The cell suspension was then transferred to a gelatin/fibronectin coated well containing 2 ml fresh medium. Adipocytes were used 2 d post transfection for experiments. Transfection efficiency ranged from 20–60% depending on the plasmid used.

### Immunofluorescence Microscopy

3T3-L1 adipocytes grown on coverslips were fixed with 4% PFA at room temperature and permeabilized with PBS containing 0,01% saponin, 0,2% gelatin and 0,02% sodium azide. After blocking with PBS containing 1% BSA and 0.2% gelatin, cells were stained with indicated antibodies overnight at 4°C. The coverslips were mounted using Mowiol (Calbiochem, Germany). Images were acquired on a Leica TCS SP2 confocal microscope (Leica Microsystems, Wetzlar, Germany) and arranged with Adobe Photoshop (Adobe Systems, Mountain View, CA).

### Subcellular Fractionation

Fractionation of 3T3-L1 adipocytes was done as described previously [38], based on [39]. Adipocytes (4×10 cm diameter Petri dishes per condition) were starved in serum-free medium and incubated with or without 1 µg/ml insulin for 30 min. After washing 3x with ice-cold HES buffer (20mM HEPES, 1 mM EDTA, 255 mM sucrose, pH 7.4), cells were collected by scraping in HES buffer containing protease inhibitors (1 mM phenylmethylsulfonyl fluoride, 10 mg/ml pepstatin, 10 mg/ml aprotinin, 5 mg/ml leupeptin) and homogenized by 10x passing through a 22 gauge needle. All following steps were performed at 4°C. The homogenate was centrifuged at 16,000 g in a F34-6-38 rotor (Eppendorff, Germany) for 30 min. The supernatant of this step was recentrifuged in a Ti70 rotor (Beckman-Coulter, Germany) at 41,000 g for 20 min. The yielded pellet was designated the high-density membrane (HDM) fraction and the supernatant was centrifuged at 180,000 g in a Ti70 rotor for 75 min. This resulted in a pellet containing the low-density membrane (LDM) fraction. The pellet from the initial centrifugation at 16,000 g was resuspended in HES puffer and centrifuged again at 16,000 g in a F34-6-38 rotor for 30 min. The resulting pellet was resuspended in HES and layered on top of a sucrose cushion (38.5% sucrose, 20 mM HEPES, 1 mM EDTA, pH 7) and centrifuged in a SW41 swing-out rotor (Beckman-Coulter, Germany) at 100,000 g for 60 min. The brownish pellet yielded contained the mitochondrial fraction (Mito). The interface containing the plasma membrane fraction (PM) was carefully removed, resuspended in HES, and centrifuged in a Ti70 rotor at 40,000 g for 20 min to obtain the pellet. All pellets were resuspended in sample buffer (62 mM Tris–HCl, pH 6.8, 2% sodium dodecyl sulfate, 10% [w/v] glycerol, 1% [w/v] b-mercaptoethanol), boiled for 5 min at 95°C and stored at −20°C. Total protein of each sample was measured by the BCA method.

### Acyl-CoA Synthetase Activity

Oleoyl-CoA synthetase activity was determined from cell lysates as described [13]. Briefly, cells were lysed for 30 minutes on ice with 1% Triton X-100, 130 mM KCl, 25 mM Tris-HCl, pH 7.4. Lysates were incubated for 10 minutes at 30°C in reaction mix containing 100 mM Tris pH 7.4, 5 mM MgCl2, 200 µM dithiothreitol, 10 mM ATP, 0.2% Triton X-100, 20 µM [^3^H]-oleate (specific activity 10 Ci/mol, bound to BSA in a 4∶1 ratio) and 200 µM CoA. After stopping with Dole’s solution (isopropanol: n-heptane: H_2_SO_4_ 40:10∶1), unreacted oleate was extracted 4x with n-heptane and the remaining oleoyl-CoA in the aqueous phase determined by scintillation counting. Background was determined with parallel samples without CoA. The results were normalized for proteins levels measured in cell lysates.

### Western Blot Analysis

For each membrane fraction, 2.5% of the original amount of proteins obtained from the subcellular fractionation was loaded and separated on a 8% SDS–polyacrylamide gel. After electrophoresis, proteins were transferred to nitrocellulose membranes. Equal loading and transfer of samples were verified by Ponceau S staining. The membranes were blocked in 5% milk powder in TBS-Tween (50 mM Tris–HCl, pH 7.4, 138 mM NaCl, 2.7 KCl, 0.1% Tween-20) for 30 min and then incubated with primary antibodies in blocking buffer containing 5% milk powder. The membranes were then washed, incubated with horseradish peroxidase-conjugated secondary antibodies and the reaction was detected with an enhanced chemiluminescence system (Amersham Life Science, Buckinghamshire, UK). Quantification of Western blots was done with ImageJ 1.37v software (Wayne Rasband, NIH).

## Results

The acyl-CoA-synthetases FATP1, ACSVL4/FATP4 and ACSL1 have distinct functions in fatty acid metabolism which are possibly mediated by their specific subcellular localization [7], [40]. However, the latter is insufficiently defined in adipocytes, especially with respect to possible changes of localization under insulin treatment. Thus, we chose to overexpress FATP1, ACSVL4/FATP4 and ACLS1-FLAG in the common 3T3-L1 adipocyte model [11], [18] in order to analyze their subcellular localization. In addition, we investigated the effects of insulin on FATP1 and ACSVL4/FATP4 localization.

### Stable Overexpression of FATP1, ACSVL4/FATP4 and ACSL1-FLAG in 3T3-L1 Cells

**Figure 1:**
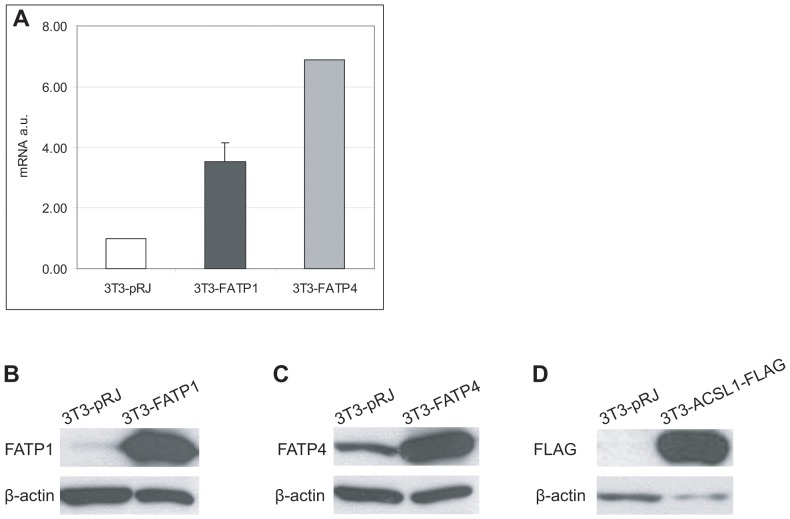
Characterization of FATP1 and ACSVL4/FATP4 expression in 3T3-L1 adipocytes. (A) Quantification of FATP1 and ACSVL4/FATP4 mRNA levels in overexpressing (3T3-FATP1, dark grey and 3T3-FATP4, light grey) and control adipocytes (3T3-pRJ, white bars) by efficiency corrected quantitative real-time PCR relative to general transcription factor 2b (Gtf2b). The relative increase of FATP1 mRNA level is 3.5-fold in 3T3-FATP1 (n = 2) and the increase of ACSVL4/FATP4 mRNA level is 6.9-fold in 3T3-FATP4 (n = 1) compared to 3T3-pRJ. (B, C, D) Analysis of protein expression by Western blotting of total cell lysates from FATP1, ACSVL4/FATP4 and ACSL1-FLAG overexpressing and control adipocytes. Densitometry indicates 11-fold overexpression of FATP1 (n = 1) and 9.0-fold overexpression of ACSVL4/FATP4 (n = 2).

We decided to use a gain of function approach to characterize the function of the most relevant acyl-CoA-synthetases of adipocytes. For this purpose, we used a retroviral transfection system with subsequent antibiotic selection to yield a pool of 3T3-L1 cells expressing the respective enzymes. Expression levels were evaluated by quantitative PCR and Western blotting. As seen in Fig. 1, FATP1 and ACSVL4/FATP4 are also endogenously expressed in 3T3-L1 adipocytes transfected with the control vector. Adipocytes overexpressing FATP1 (3T3-FATP1) or ACSVL4/FATP4 (3T3-ACSVL4/FATP4) show a marked increase in mRNA (Fig. 1A) and protein content (Fig. 1B, C) compared to control transfected cells (3T3-pRJ). The relative increase in protein level was higher for FATP1 than ACSVL4/FATP4. The presence of ACSL1 (3T3-ACSL1-FLAG) was verified by Western blotting using anti-FLAG antibodies.

### FATP1 and ACSVL4/FATP4 are Localized in the Endoplasmic Reticulum

The exact knowledge of the subcellular localization of FATP1 and ACSVL4/FATP4 is necessary for understanding their function, especially in light of the controversies over the fundamental mechanisms of fatty acid transport. Here, we focused on immunofluorescence microscopy to analyze the localization of FATP1 and ACSVL4/FATP4.

**Figure 2:**
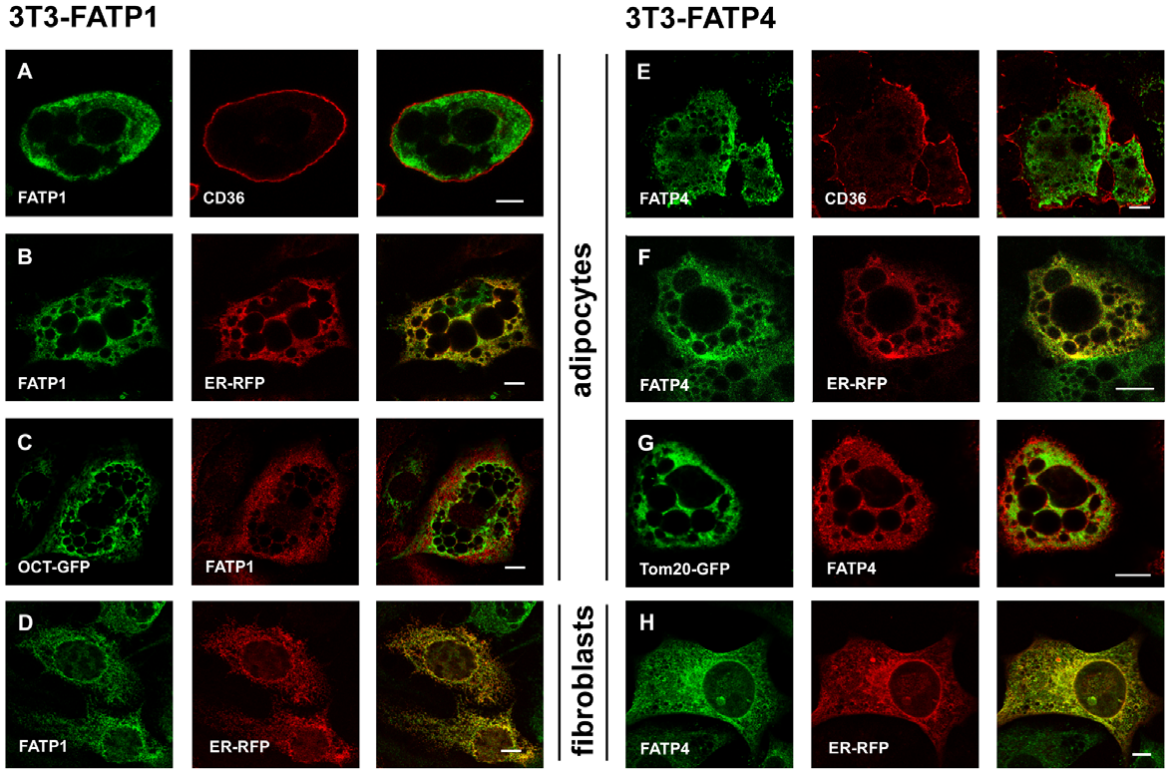
FATP1 and ACSVL4/FATP4 are localized to the endoplasmic reticulum in 3T3-L1 adipocytes. (A–D) Comparative analysis of the intracellular localization of FATP1. FATP1 (green: A, B + D/red: C; affinity purified rabbit anti FATP1) does not overlap with the plasma membrane marker CD36 (A) (red, mouse polyclonal anti CD36) or the mitochondrial marker ornithin-carbomoyl-transferase (C) (green, OCT-GFP; C-terminal coupled with GFP, expressed by nucleofection). FATP1 (green) co-localizes with ER marker Sec61β (red; C-terminal coupled with RFP, expressed by nucleofection) in adipocytes (B) and fibroblasts (D) as indicated by the yellow color in the overlap image. In adipocytes, a distinct area is covered by FATP1 but not ER (B). Representative images from single confocal sections are shown and dimension bars are 10 µm for all images. (E–H) Comparative analysis of the intracellular localization of ACSVL4/FATP4. ACSVL4/FATP4 (green: E, F + H/red: G); affinity purified rabbit anti FATP4) does not co-localize with CD36 (E) (red) or Tom20 (G) (green). ACSVL4/FATP4 (green) overlaps with Sec61β-RFP (red) in both adipocytes (F) and fibroblasts (H).

We combined immunofluorescence staining and expression of fluorescent organelle markers to investigate the localization via confocal laser scanning microscopy. As seen in Fig. 2A, FATP1 shows a reticular intracellular pattern that spreads over the cell in both fibroblasts (Fig. 2D) and adipocytes (Fig. 2A). We used antibodies against endogenous CD36 to stain the plasma membrane [41]. Our results demonstrate that FATP1 is present in close proximity of the plasma membrane, but does not overlap with CD36 (Fig. 2A). The mitochondrial marker OCT-GFP displays a pattern that is distinctively different from FATP1 (Fig 2C). In contrast, FATP1 shares the same localization with the ER marker protein Sec61β [42] (Fig. 2A,D), with the exception of a small area that is covered by FATP1 only (Fig. 2C). The distribution of ACSVL4/FATP4 is very similar to FATP1 (Fig 2E–H). Again, a pattern can be identified that is most compatible with the ER-Marker (Fig. 2F+H), but not with CD36 (Fig. 2E) or the mitochondrial marker Tom20-GFP (Fig. 2G). The localization of ACSVL4/FATP4 on the ER is especially obvious when looking at fibroblasts (Fig. 2H). Here, a complete overlap between ACSVL4/FATP4 and the ER-marker can be observed.

### ACSL1 is Localized on Mitochondria

We analyzed the subcellular localization of ACSL1, an acyl-CoA-synthase that can drive fatty acid uptake [13], [43].

**Figure 3:**
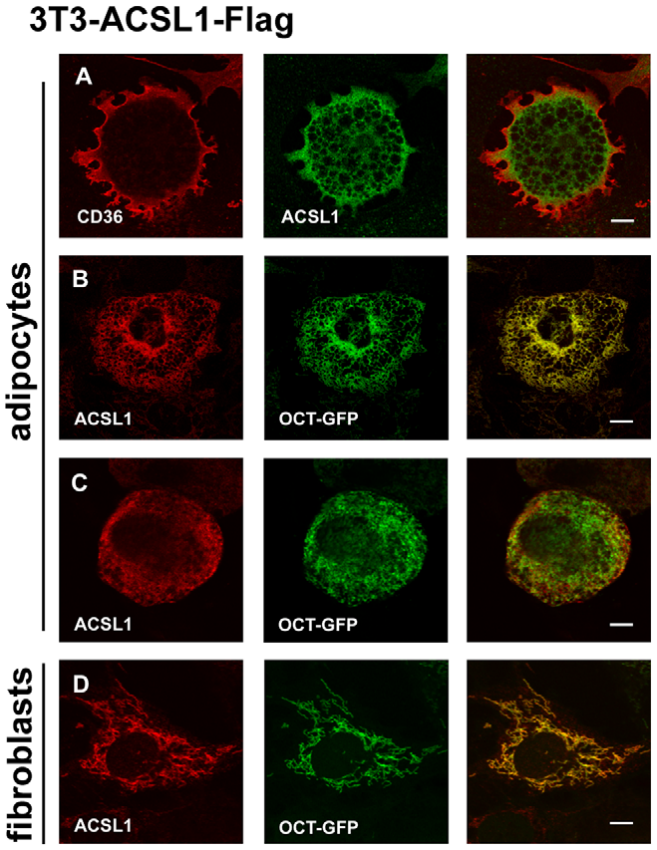
ACSL1-FLAG is localized to mitochondria in 3T3-L1 adipocytes. (A–D) Comparative analysis of the intracellular localization of FLAG -tagged ACSL1. (A) ACSL1-FLAG (green); mouse monoclonal anti FLAG M2) does not co-localize with CD36. (red). (B–C) Congruency of ASCL1-FLAG (red) with the mitochondrial marker OCT-GFP is excellent for lowly differentiated (B) but less complete for highly differentiated adipocytes (C). (D) In fibroblasts, ACSL1-FLAG overlaps completely with the mitochondrial marker.

Our data show that ACSL1-FLAG is located on organelles with a worm-shaped pattern that is typical for mitochondria. This morphology is most striking at the stage of fibroblasts, when ACSL1-FLAG completely co-localizes with the mitochondrial marker GFP-OCT (Fig. 3D). While this characteristic mitochondrial morphology is still found at the early stage of adipocyte differentiation (Fig. 3B), it is replaced by a more fragmented and diffuse distribution (Fig. 3A+C) in highly differentiated cells with a less impressive marker co-localization (Fig. 3C). This change of mitochondria morphology through adipocytes differentiation is known for 3T3-L1 cells [44]. Although a localization of ACSL1 on the plasma membrane was reported [29], [45], we could not find a co-localization with CD36 (Fig. 3A).

### FATP1, ACSVL4/FATP4 and ACSL1-FLAG Overexpressing 3T3-L1 Adipocytes Show Increased Fatty Acid Activation and Uptake

**Figure 4:**
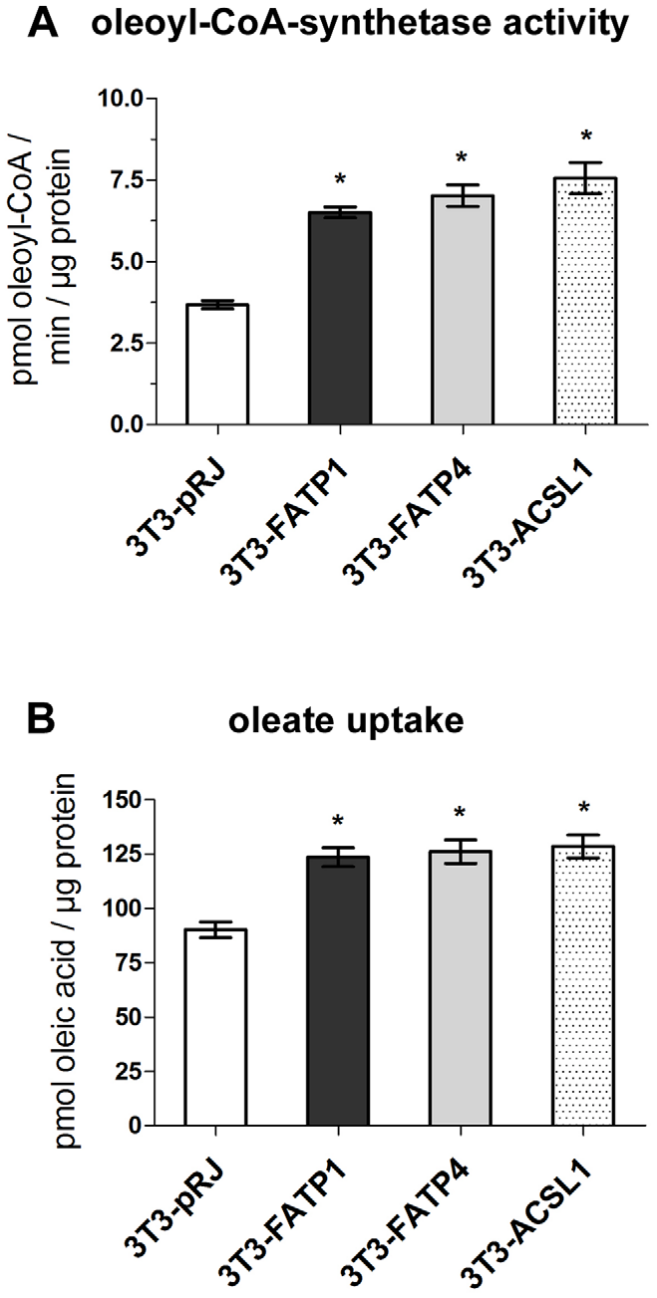
FATP1, ACSVL4/FATP4 and ACSL1-FLAG overexpression enhances fatty acid activation and uptake. (A) Oleoyl-CoA synthetase activity was determined from cell lysates of 3T3-pRJ control (white bars), 3T3-FATP1 (light grey), 3T3-FATP4 (dark grey) and 3T3-ACSL1-FLAG adipocytes (punctate) for ten minutes in the presence of 20 µM [^3^H]-oleate (specific activity 10 Ci/mol). * p<0.05 vs 3T3-pRJ; n = 4. (B) Oleate uptake was measured by incubating adipocytes for 3 h with 200 µM [^3^H]-oleic acid bound to 100 µM fatty acid free BSA (specific activity 0.5 Ci/mol). * p<0.05 vs 3T3-pRJ; n = 4.

To confirm that overexpression of FATP1, ACSVL4/FATP4 and ACSL1-FLAG results in functional acyl-CoA-synthetases, we measured the oleoyl-CoA-synthetase activity from lysates of the corresponding cell lines. Our results show that FATP1, ACSVL4/FATP4 and ACSL1-FLAG are functional because the total enzyme activity is enhanced in all cell lines (Fig. 4A). This increased enzyme activity also translates into a significantly higher fatty acid uptake rate, as compared to control cells (Fig. 4B). These results show that overexpression of all three intracellular acyl-CoA-synthetases enhances fatty acid uptake in our model system.

### Insulin Increases Fatty Acid Uptake without Changing Localization of FATP1 or ACSVL4/FATP4

Insulin is known to increase fatty acid uptake in adipocytes and a translocation of FATP1 was considered as the key mechanism behind this effect [11]. However, recent papers have observed that FATP1 is localized on mitochondria and fulfills several metabolic functions that do not require localization to the plasma membrane [19], [20].

**Figure 5:**
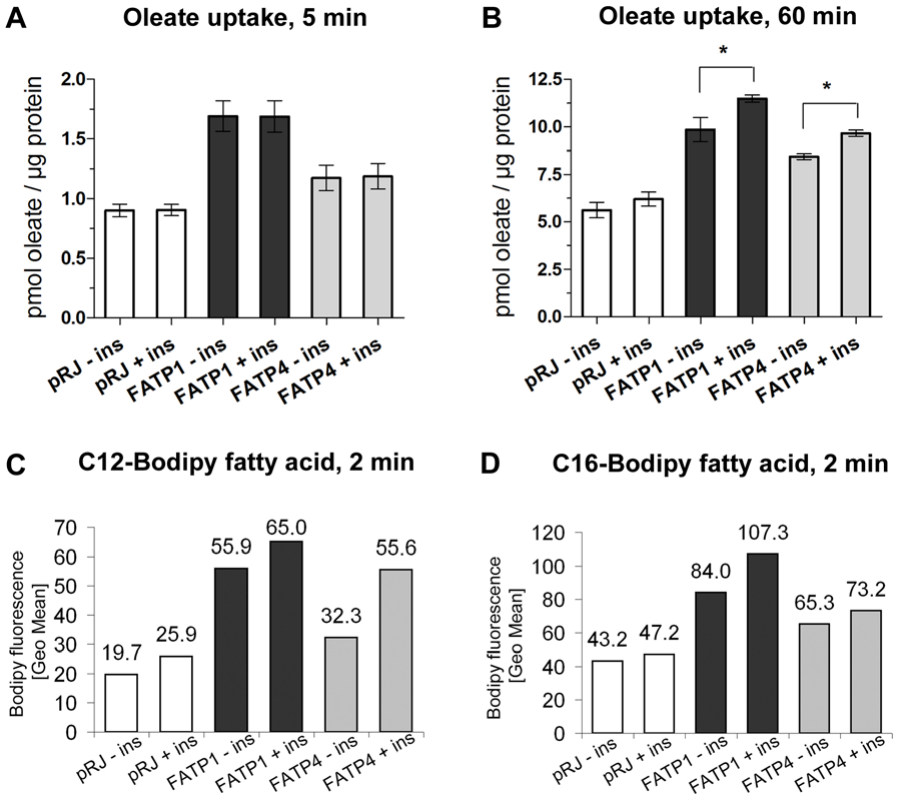
Short term incubation with insulin enhances uptake of fluorescent fatty acids. (A+B) 3T3-FATP1 (dark grey bars), 3T3-FATP4 (light grey bars) and 3T3-pRJ control adipocytes (white bars) were pretreated with or without 1 µg/ml insulin for 10 min, followed by co-incubation with the with 340 µM [^3^H]-oleic acid bound to 170 µM fatty acid free BSA (specific activity 1 Ci/mol) for 5 min (A) or 170 µM [^3^H]-oleic acid bound to 85 µM fatty acid free BSA (specific activity 1 Ci/mol) for 60 min (B). * p<0,05; n = 3. (C+D) 3T3-FATP1 (dark grey bars), 3T3-FATP4 (light grey bars) and 3T3-pRJ control adipocytes (white bars) were pretreated with or without 1 µg/ml insulin for 18 min, followed by co-incubation with 2 µM Bodipy fatty acids (FA_By12_ (C); n = 3/FA_By16_ (D); n = 2) bound to 2 µM BSA for 2 min. Subsequent FACS analysis shows a more pronounced increase in geographical mean fluorescence signal after insulin treatment for 3T3-FATP1 (dark grey bars) and 3T3-FATP4 (light grey bars) compared to 3T3-pRJ control adipocytes (white bars), which is however restricted to specific Bodipy fatty acid species (FA_By12_ for 3T3-FATP4 (C) and FA_By16_ for 3T3-FATP1 (D)). Representative experiments are shown.

We first measured the uptake of the physiological substrate oleate for 5 min, but could not detect an increase in uptake rate after insulin treatment (Fig. 5A). Extending the incubation time of insulin and oleate to 1 h resulted in a significant increase of uptake for 3T3-FATP1 and ACSVL4/FATP4, but not for 3T3-pRJ adipocytes (Fig. 5B). Next, we used fluorescent Bodipy fatty acids with two different hydrocarbon chain lengths (FA_By12_ and FA_By16_) to measure uptake rates. Bodipy fatty acids are metabolized similar to naturally occurring long chain fatty acid species, but are reported to have a higher affinity towards FATP1 and ACSVL4/FATP4 [46]. They have been frequently used to measure fatty acid uptake [11], [13], [16], [43]. By using them, we aimed to investigate the role of both isoforms in insulin-mediated fatty acid uptake more selectively. Our results confirm that insulin increases the uptake of fluorescent fatty acids in adipocytes in general (Fig. 5C–D). However, compared to control adipocytes, the effect of insulin on the uptake rate was more pronounced for FA_By12_ in 3T3-FATP4 and for FA_By16_ in 3T3-FATP1. This observation indicates that both FATP1 and ACSVL4/FATP4 contribute to insulin-mediated fatty acid uptake but with different substrate specificity.

**Figure 6:**
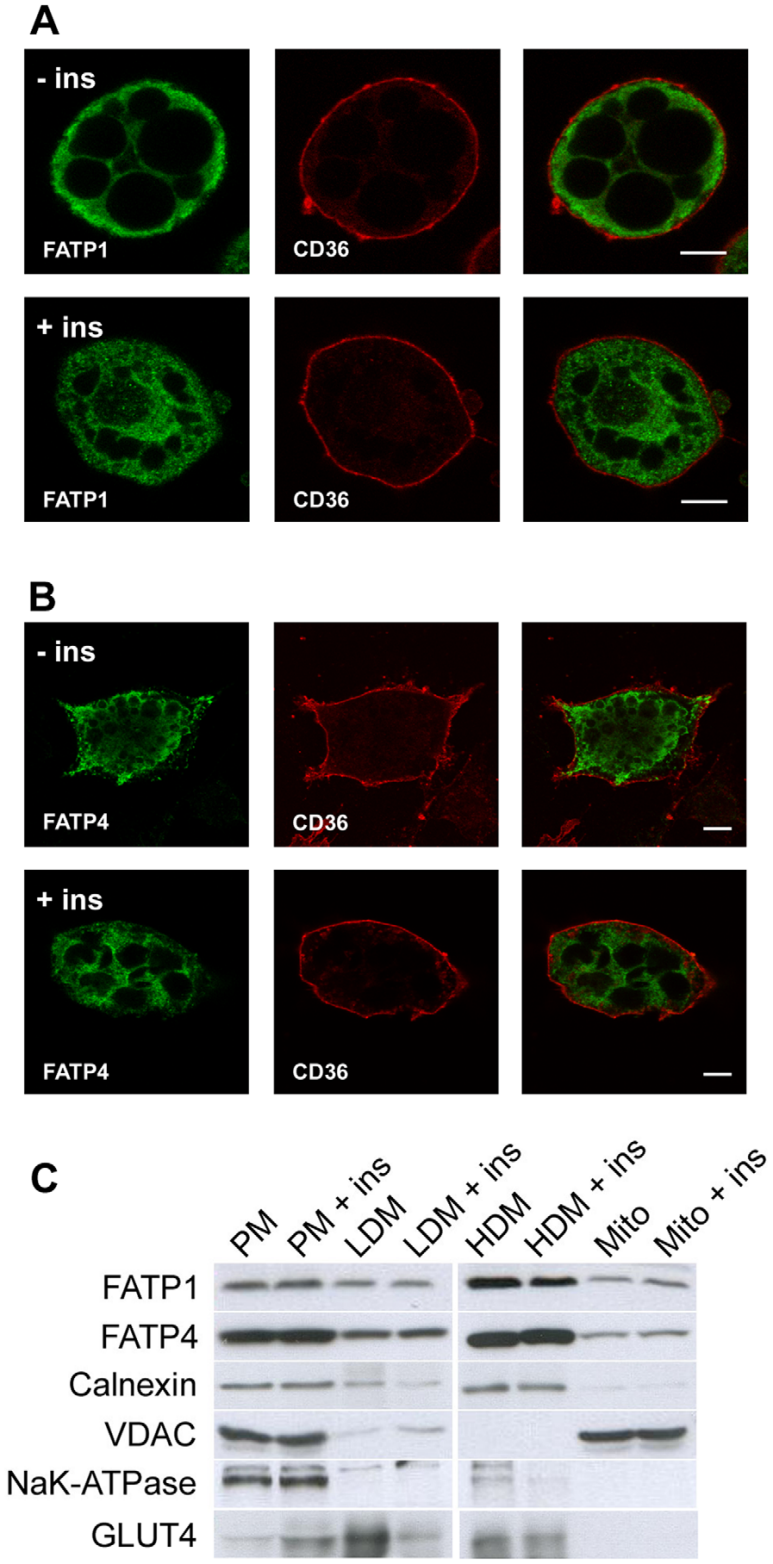
Short term incubation with insulin does not change intracellular localization of FATP1 and ACSVL4/FATP4. (A+B) 3T3-FATP1 and 3T3-FATP4 were treated or non treated for 20 min with 1 µg/ml insulin, stained and analyzed for changes of localization with confocal laser scanning microscopy. No co-localization of FATP1 (A) or ACSVL4/FATP4 (B) with the plasma membrane localized CD36 was observed, neither for insulin treated nor for non treated cells. (C) Subcellular fractionation of wild-type 3T3-L1 adipocytes treated or non treated for 20 min with 1 µg/ml insulin. 20 µg of each subcellular fraction were applied. Upon insulin stimulation, a significant increase in GLUT4 was observed in the plasma membrane (PM) fraction with concomitant reduction in the low (LDM) and high density membrane fraction (HDM). This shift was not observed for FATP1 and ACSVL4/FATP4. FATP1, ACSVL4/FATP4 and the ER marker calnexin share the same distribution pattern. This pattern differs from the distribution of both, voltage-dependent anion-selective channel protein 1 (VDAC) that is localized primarily on mitochondria and partly on the plasma membrane and the plasma membrane localized sodium-potassium ATPase. Representative blots are shown.

To investigate whether insulin induces a change of localization of FATP1 or ACSVL4/FATP4, we first used immunofluorescence microscopy. As seen in Fig. 6A–B, neither FATP1 nor ACSVL4/FATP4 showed co-localization with the plasma membrane marker CD36 upon insulin stimulation for 20 min. This was also investigated after 60 min of insulin treatment, but the staining patterns remained unchanged (T.Z. and J.F., not shown). In order to confirm these findings, we used subcellular fractionation of wildtype, non-transfected 3T3-L1 adipocytes as a second, independent approach (Fig. 6C). We could observe a clear shift of GLUT4 from the LDM/HDM fraction to the PM fraction upon insulin stimulation, but did not detect a similar change for FATP1 or ACSVL4/FATP4. Interestingly, we found that FATP1 and ACSVL4/FATP4 are present in all membrane fractions, which was also observed by others [11]. However, most fractionation protocols for adipocytes are optimized for GLUT4 translocation, but not for a clear-cut discrimination between subcellular compartments. To overcome this problem, we decided to compare the distribution of FATP1 and ACSVL4/FATP4 with those of marker proteins. We used calnexin as a marker for the ER [47], voltage-dependent anion-selective channel protein 1 (VDAC1) for mitochondria [48] and Na^+^/K^+^-ATPase for the plasma membrane [49]. Remarkably, calnexin has the same distribution pattern as FATP1 and ACSVL4/FATP4. In contrast, VDAC1 is predominantly found on mitochondria and partly also on the plasma membrane, which is in line with its reported localization on both organelles [48]. The signal for Na+/K+-ATPase is very weak and found only in the plasma membrane and mitochondrial fraction. These results show that FATP1 and ACSVL4/FATP4 are most likely localized in the ER, which is consistent with our data from immunofluorescence microscopy.

In conclusion, our results show that short term incubation with insulin increases fatty acid uptake, but without changing the intracellular localization of FATP1 and ACSVL4/FATP4.

### Insulin-mediated Glucose Uptake is Increased in FATP1 and ACSVL4/FATP4 Overexpressing 3T3-L1 Adipocytes

**Figure 7:**
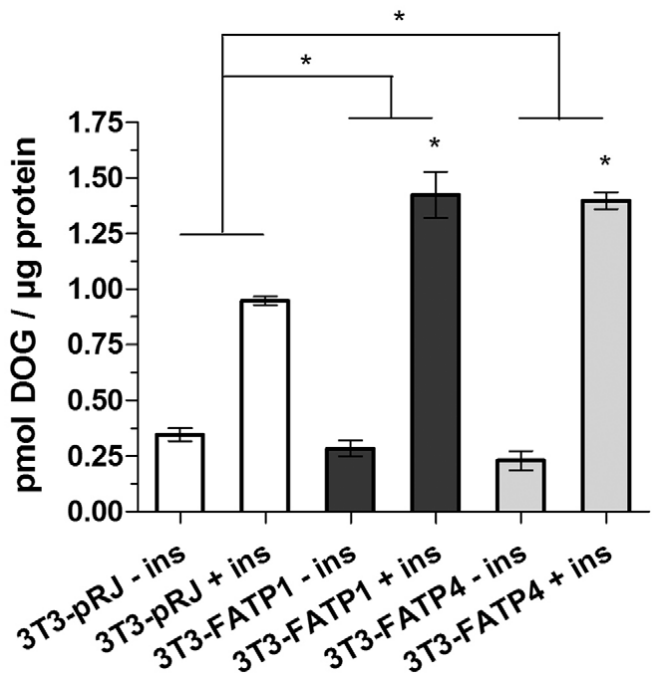
Insulin-mediated glucose uptake is enhanced in FATP1 and ACSVL4/FATP4 overexpressing adipocytes. 3T3-FATP1 (dark grey bars) and 3T3-FATP4 adipocytes (light grey bars) and control adipo-cytes (white bars) were incubated with 1 µg/ml insulin for 20 min followed by 10 min of co-incubation with 0.1 mM 2-deoxy-D-glucose [DOG], 1 mCi/ml. Glucose uptake is significantly higher for insulin treated cells (* p<0,05) and this effect is more pronounced in FATP1 and ACSVL4/FATP4 overexpressing adipocytes (* p<0,05), n = 3.

Glucose and fatty acid metabolism are intimately connected. Therefore we investigated whether overexpression of FATP1 and ACSVL4/FATP4 has an impact on insulin-mediated glucose uptake. The basal glucose uptake rate was not significantly different between 3T3-FATP1, 3T3-FATP4 or the control adipocytes. However, we found that the increase in glucose uptake upon insulin stimulation was significantly higher in 3T3-FATP1 (5.8-fold) and 3T3-FATP4 (7.6-fold) as compared to control adipocytes (2.9-fold), as shown in Fig. 7.

## Discussion

With our work, we sought to resolve some of the ongoing controversies about the mechanism of FATP-mediated fatty acid transport. Our approach was to focus on identifying the subcellular localization of FATP1, ACSVL4/FATP4 and the acyl-CoA-synthetase ACSL1. We selected adipocytes as our model system because they are highly relevant for the pathogenesis of diseases while the localization of FATP1, ACSVL4/FATP4 and ACSL1 is poorly defined in this cell line.

Unlike previous studies, we chose to overexpress FATP1 and ACSVL4/FATP4 instead of knockdown by RNAi [18]. Multiple acyl-CoA synthetases are coexpressed in adipocytes [50] and depletion of one enzyme might be compensated by the remaining isoforms. The overexpression of FATP1 and ACSVL4/FATP4 was successful as determined by both quantitative PCR and Western Blotting (Fig. 1). The increases in acyl-CoA synthetase activity (1.9-fold for FATP1 and ACSVL4/FATP4; Fig. 4A) and oleate uptake (1.23-fold for FATP1 and 1.24-fold for ACSVL4/FATP4; Fig. 4B) were less than what we initially expected based on the fold overexpression. The endogenous levels of FATP1 and ACSVL4/FATP4 are low comparative to other acyl-CoA-synthetases such as ACSL1, which is highly expressed in adipocytes [9], [25]. Thus, although overexpression leads to a significant increase of a single FATP relative to its endogenous level, this effect is much less pronounced when looking at the total cellular acyl-CoA synthetase activity. In addition, fatty acid uptake is the net result of multiple processes involving several different proteins [5]. Besides the acyl-CoA synthetases/FATPs, upstream proteins like fatty acid binding proteins [51] or caveolin [52], and downstream enzymes such as glycerol-3-phosphate acyltransferase [53] or 1-acyl-glycerol-3-phosphate acyltransferase [54] influence the extent of fatty acid uptake as well. Therefore, the overexpression of acyl-CoA synthetases/FATPs is only able to increase fatty acid uptake until other enzymes/proteins become rate limiting. Finally, we do not have any indications for secondary effects caused by our stable expression approach but did not systematically investigate this. It is possible that e.g. increased intracellular fatty acyl-CoA concentrations may have changed the activity of transcription factors, and ultimately the extent of fatty acid uptake. However, in hepatocytes only ACSL3 but none of the other acyl-CoA synthetases/FATPs influenced lipogenic transcription factors suggesting this is not a general concern.

We localized ACSVL4/FATP4 to the ER of 3T3-L1 adipocytes. This is consistent with previous studies that identified the same location for ACSVL4/FATP4 in different cell lines [13], [14], [23]. While the acceptance of ACSVL4/FATP4 as an ER protein is growing, the localization of FATP1 remains controversial. Interestingly, we found that FATP1 has a very similar distribution pattern like ACSVL4/FATP4. An intracellular localization has been described previously for FATP1 [11], [18], [20] which was recently proposed to represent mitochondria [19], [20]. In addition, FATP1 was reported to be at least partially associated with the plasma membrane [11], [18]. Our own results show that FATP1 is located primarily at the ER (Fig. 2), which to our knowledge has not been openly acknowledged so far. In addition, FATP1 was found on internal membranes possibly reflecting the Golgi apparatus. This localization was previously described for FATP1 in skeletal muscle cells [21]. We did not observe a significant overlap of FATP1 with mitochondrial membranes, in contrast to others [19], [20]. Regarding the functional role of FATP1, it is important to note here that we did not see an association with the plasma membrane, consistent with recent studies [19], [20].

In our opinion, two fundamental problems cause the incoherent reports concerning the localization of FATP1 and ACSVL4/FATP4 in adipocytes: first, the specific morphology of this type of cells and secondly, the experimental approaches used to localize the proteins. We observed that identifying organelles by confocal laser scanning microscopy was much easier and more distinctive for 3T3-L1 fibroblasts than for differentiated adipocytes. Unlike fibroblasts that spread horizontally, adipocytes primarily grow on the vertical axis due to the high cell density required for their differentiation. This growth pattern makes the correct identification of cell structures difficult. Furthermore, adipocytes incorporate several lipid droplets that scatter laser light, which affects the focus and the resolution of confocal microscopy. A high resolution however is required as the cytoplasm is tightly compressed by lipid droplets, posing a challenge for the identification of subcellular organelles. The second problem contributing to the confusion is the experimental approach. Immunofluorescence microscopy is limited by the drawbacks mentioned above. Another common approach is subcellular fractionation. Most fractionation protocols for adipocytes are optimized for GLUT4 translocation, but lack the specificity and resolution required for a clear-cut distinction between different cellular compartments. Similar to our own results, other groups found FATP1 [11] or ACSL1 [45] across all four fractions (designated plasma membrane, LDM, HDM and mitochondrial fraction), which is in contrast to results derived from microscopy. To overcome this problem, we compared the distribution patterns of FATP1, ACSVL4/FATP4 and ACSL1 with several marker proteins (Fig. 2). The results we obtained were consistent with our microscopy data. Nevertheless, our results have to be viewed in light of the methodological limitations mentioned above.

Our results also show that the intracellular localization of overexpressed FATP1, ACSVL4/FATP4 and ACSL1 is sufficient to significantly enhance basal fatty acid uptake. This observation was already made by us and others for ACSVL4/FATP4 [13], [23] and is not compatible with the idea that FATPs are plasma membrane bound transporter proteins [6], [55]. Instead, our results favor a model in which the influx of fatty acids is driven by a concentration gradient arising from the conversion of intracellular fatty acids to acyl-CoAs [7], [56]. This interpretation is further supported by our experiments with insulin stimulated fatty acid uptake.

Insulin has been shown before to enhance the fatty acid uptake of adipocytes and other cell types. Our results support this observation even if the magnitude measured now is at the lower end compared to previous data. Interestingly, we found a significant increase of oleate uptake after a 60 min incubation with insulin and fatty acids but not after 15 min (Fig. 5 A, B). This is also reflected by the different incubation conditions found in other studies, where an effect of insulin on oleate uptake was shown [18], [57]. For the increased uptake of fluorescent Bodipy fatty acids 20 min of insulin treatment were sufficient (Fig 5 C, D). Bodipy fatty acids are preferentially metabolized by FATPs as compared to other long chain acyl-CoA synthetases [46], and the effects of FATP overexpression are therefore more pronounced when using Bodipy fatty acids.

There are conflicting ideas about the molecular mechanism by which insulin enhances fatty acid uptake [14], [16], [58]. In this work, we focused on the localization and did not observe a translocation of FATP1 or ACSVL4/FATP4 from intracellular membranes to the cell surface (Fig. 6). This contradicts publications that proposed the translocation of FATP1 as the main mechanism [11], [16], but is supported by recent studies which also failed to observe a translocation [19], [20]. We used two independent experimental approaches to investigate the localization of FATP1 and ACSVL4/FATP4 under conditions where the uptake of fluorescent fatty acids was already increased by insulin (20 min). Therefore, we argue that a translocation of FATP1 is not a prerequisite for the enhancement of either basal or insulin mediated fatty acid uptake since the intracellular localization of FATP1 appears sufficient to ensure both. It is thus necessary to consider other models of insulin action. We did not observe a change in the localization of CD36 after insulin treatment, as was described for muscle cells [59], [60]. Almost all CD36 was already at the plasma membrane under non stimulated conditions (Fig. 2 A, E).

If FATP1 and ACSVL4/FATP4 are not insulin sensitive fatty acid transporter proteins, and the localization of CD36 at the plasma membrane of adipocytes remains undisturbed, then how is the effect of insulin mediated? At present, we are not able to answer this question satisfyingly. A very recent discovery was that the enzyme activity of ACSVL4/FATP4 stably expressed in C2C12 muscle cells was increased in a manner dependent on insulin signaling [14]. Increased acyl-CoA synthetase activity has been repeatedly shown to result in enhanced fatty acid uptake [56]. Moreover another biosynthetic enzyme, glycerol-3-phosphate acyl transferase is also regulated by insulin (reviewed by [61]). This would be in line with the concept that intracellular fatty acid and lipid metabolism contributes significantly to cellular fatty acid uptake [7]. However other possibilities remain [58], and the oleoyl-CoA synthetase activity of the adipocyte cell lines established here was not significantly changed by insulin treatment (T.Z., results not shown).

Our data also suggest that both overexpressed FATP1 and ACSVL4/FATP4 enhance the effect of insulin on fatty acid uptake (Fig. 5). This is in contrast to previous knockdown/knockout studies which suggested that only FATP1 has a significant role in insulin-mediated fatty uptake [11], [18] [16]. We think that the coverage of insulin-mediated fatty acid uptake by two proteins of the same family is likely of physiological relevance because serum lipids contain a wide spectrum of fatty acid species with varying carbon chain lengths. FATP1 and ACSVL4/FATP4 differ in their substrate specificities [62], which is also reflected by our data for the uptake of Bodipy fatty acids of different chain lengths (Fig. 5 C, D). The parallel expression of both FATPs would ensure the resorption of a broader spectrum of fatty acids.

Overexpression of FATP1 and ACSVL4/FATP4 also increased insulin-mediated glucose uptake (Fig. 7). We speculated initially that FATP1 and ACSVL4/FATP4 overexpression might increase the activation of AMP-activated kinase (AMPK), which in turn could lead to an increase of GLUT4 translocation [63]. However, the phosphorylation of AMPK was actually decreased in FATP1 and ACSVL4/FATP4 overexpressing 3T3-L1 cells under basal conditions (Fig. S1). Insulin treatment decreased the activation state of AMPK even further, in agreement with published evidence [64], [65]. Thus, it remains currently unclear how FATP expression increases insulin-mediated glucose uptake.

Taken together, our results demonstrate that the intracellular localization of FATP1 and ACSVL4/FATP4 on the ER and ACSL1 on mitochondria is sufficient to enhance fatty acid uptake. Short term insulin treatment leads to an increased uptake of fluorescent fatty acids, which is more pronounced in FATP1 and ACSVL4/FATP4 overexpressing adipocytes, but not accompanied by a change of localization of either protein.

Our interpretation is that the FATP acyl-CoA synthetases are metabolically trapping intracellular fatty acids, and through this mechanism contribute to the efficiency of insulin mediated fatty acid uptake. It remains for future studies to determine if the adipocyte FATP acyl-CoA synthetases themselves are activated by insulin, or if they support insulin mediated fatty acid uptake more indirectly.

## Supporting Information

Figure S1 **Phosphorylation of AMPK after insulin treatment.** 3T3-FATP1, 3T3-FATP4 and control adipocytes (pRJ) were incubated with 1.0 µg/ml insulin for 20 min, as in Fig. 7. Western blotting of total cell lysates was with antibodies against total (AMPK) and phosphorylated (P-AMPK; T172) AMP-activated protein kinase.
(TIF)
