# Supplementary figures and images for: Overexpressed FATP1, ACSVL4/FATP4 and ACSL1 Increase the Cellular Fatty Acid Uptake of 3T3-L1 Adipocytes but Are Localized on Intracellular Membranes
Source: PLoS One. 2012 Sep 14;7(9):e45087. doi: 10.1371/journal.pone.0045087 (PMC3443241; doi:10.1371/journal.pone.0045087)

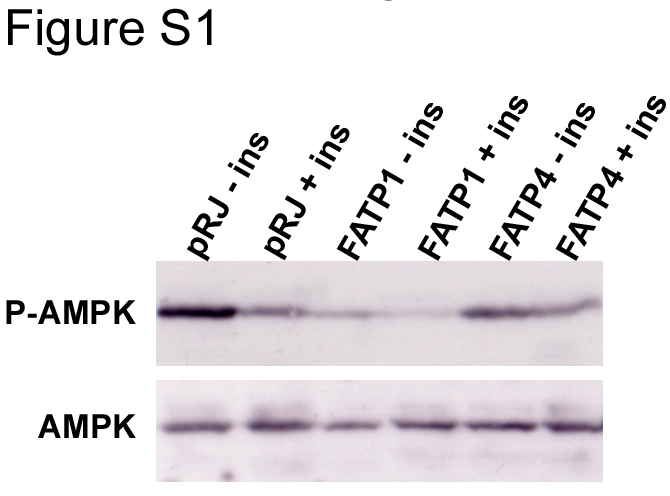

Supplement: Figure S1 — Phosphorylation of AMPK after insulin treatment. 3T3-FATP1, 3T3-FATP4 and control adipocytes (pRJ) were incubated with 1.0 µg/ml insulin for 20 min, as in Fig. 7. Western blotting of total cell lysates was with antibodies against total (AMPK) and phosphorylated (P-AMPK; T172) AMP-activated protein kinase. (TIF) [file pone.0045087.s001.tif]
